# TRAM Is Involved in IL-18 Signaling and Functions as a Sorting Adaptor for MyD88

**DOI:** 10.1371/journal.pone.0038423

**Published:** 2012-06-07

**Authors:** Hidenori Ohnishi, Hidehito Tochio, Zenichiro Kato, Norio Kawamoto, Takeshi Kimura, Kazuo Kubota, Takahiro Yamamoto, Tatsuyoshi Funasaka, Hiroshi Nakano, Richard W. Wong, Masahiro Shirakawa, Naomi Kondo

**Affiliations:** 1 Department of Pediatrics, Graduate School of Medicine, Gifu University, Gifu, Japan; 2 Department of Molecular Engineering, Graduate School of Engineering, Kyoto University, Kyoto, Japan; 3 Laboratory of Molecular and Cellular Biology, Department of Biology, School of Natural System, Kanazawa University, Kakuma-machi, Kanazawa, Japan; University of Pittsburgh, United States of America

## Abstract

MyD88, a Toll/interleukin-1 receptor homology (TIR) domain-containing adaptor protein, mediates signals from the Toll-like receptors (TLR) or IL-1/IL-18 receptors to downstream kinases. In MyD88-dependent TLR4 signaling, the function of MyD88 is enhanced by another TIR domain-containing adaptor, Mal/TIRAP, which brings MyD88 to the plasma membrane and promotes its interaction with the cytosolic region of TLR4. Hence, Mal is recognized as the “sorting adaptor” for MyD88. In this study, a direct interaction between MyD88-TIR and another membrane-sorting adaptor, TRAM/TICAM-2, was demonstrated *in vitro*. Cell-based assays including RNA interference experiments and TRAM deficient mice revealed that the interplay between MyD88 and TRAM in cells is important in mediating IL-18 signal transduction. Live cell imaging further demonstrated the co-localized accumulation of MyD88 and TRAM in the membrane regions in HEK293 cells. These findings suggest that TRAM serves as the sorting adaptor for MyD88 in IL-18 signaling, which then facilitates the signal transduction. The binding sites for TRAM are located in the TIR domain of MyD88 and actually overlap with the binding sites for Mal. MyD88, the multifunctional signaling adaptor that works together with most of the TLR members and with the IL-1/IL-18 receptors, can interact with two distinct sorting adaptors, TRAM and Mal, in a conserved manner in a distinct context.

## Introduction

Toll-like receptors (TLRs) are representative innate immune receptors that recognize pathogen-associated molecular patterns (PAMPs). MyD88, a cytosolic adaptor protein, is involved in the signaling pathways initiated by all of the reported TLRs with the exception of TLR3 [1]. Usually, PAMPs first bind to the extracellular domain of the TLRs, and the cytosolic region of TLRs then interact with MyD88, which allows the signal to be transmitted to the downstream kinase Interleukin (IL) -1 receptor associated kinase 4 (IRAK4). The resulting activation of IRAK4 eventually leads to the activation of the transcription factors NF-κB and AP-1 via conserved phosphorylation cascades [2]. MyD88 is composed of two functional domains: an N-terminal death domain (DD) and a C-terminal Toll/Interleukin-1 receptor homology (TIR) domain [3]. The DD is a protein interaction module that is involved in a variety of cellular events. Similar to the DD, the TIR domain also mediates protein-protein interactions via homotypic TIR-TIR interactions. In contrast to the DD, the TIR domain is almost exclusively found in the TLR related cytosolic adaptors or in the cytosolic regions of the TLRs, IL-1 and IL-18 receptors. Homotypic interactions of these protein interaction modules play a pivotal role in transmitting the signals downstream from the TLR; the TIR of MyD88 interacts with the TIR of the TLRs, and the DD of MyD88 interacts with the DD of IRAK4, which forms a large protein complex called the Myddosome [4].

TLR4 signaling, the best characterized signaling pathway among a dozen of known TLR pathways, is activated by lipopolysaccharide (LPS) from gram-negative bacteria, and the pathway plays a major role in endotoxin shock. Two modes of the signaling have been described: the MyD88-dependent and MyD88-independent pathways. In the MyD88-dependent TLR4 signal transduction pathway, another TIR domain-containing adaptor protein, Mal (also called TIRAP), plays an important role. Mal binds MyD88 via a homotypic TIR interaction and then associates with the plasma membrane using its PIP2-binding domain. Thus, Mal has been suggested to serve as a “sorting adaptor” that recruits the “signaling adaptor”, MyD88, to the membrane region where the activated TLR4 resides [5]. Mal is not an essential factor for the signaling because signal transduction can occur even without Mal [6], [7], but Mal can substantially facilitate the signaling. A pair of TIR domain-containing adaptors, TIR domain-containing adaptor inducing IFN-β (TRIF) and TRIF-related adaptor molecule (TRAM), is known to play important roles in the MyD88-independent TLR4-signaling pathway in which TRIF and TRAM function as the signaling and the sorting adaptors, respectively. This pathway transmits signals from TLR4 at early endosomes after the LPS-induced internalization of TLR4 [8], [9]. TRAM is known to deliver TRIF to the endosomes via a specific region of the plasma membrane by using its myristoylation site and polybasic region [10]. These findings indicate that the specific combinations of the sorting and the signaling TIR-containing adaptors define the specific signal transduction pathways.

MyD88 is also involved in acquired immune responses because it mediates the signals from the inflammatory cytokines IL-1 and IL-18; ligand-activated IL-1/IL-18 receptors that subsequently interact with MyD88 to trigger downstream protein kinase cascades that eventually activate the transcription factors NF-κB and AP-1 in a similar manner to TLR signaling. Although the intracellular signaling pathway is similar to the MyD88-dependent TLR4 pathway, the sorting-adaptor Mal is not involved [11]. As mentioned above, TLR4 signaling is facilitated either by Mal (MyD88-dependent pathway) or TRAM (MyD88-independent pathway) in a pathway-dependent manner. The former mainly localizes in PIP2 rich plasma membrane regions, while the latter is found not only in the plasma membrane but also in the internalized early endosomes that dispatch the signals. Thus, different sorting adaptors recruit MyD88 to different membrane regions and create distinct types of signal initiation complexes. In contrast to TLR signals, IL-1/IL-18 signaling has not thus far been thought to require such sorting adaptors. Interestingly, Kagan *et al.* reported that an engineered MyD88 that is endowed with PIP2 binding ability could rescue the LPS-TLR4 signaling in mouse embryonic fibroblast (MEF) cells from MyD88 and Mal double knockout mice, although it failed to rescue IL-1 signaling in the cells [5]. This observation suggests that TLR4 and IL-1R are located in distinct regions of the plasma membrane, which then raises the hypothesis that unidentified sorting adaptors selectively bring MyD88 to the appropriate membrane region to form signal initiation complexes with activated IL-1 and IL-18 receptors.

In this study, we sought the sorting adaptor for IL-18 signaling and discovered that TRAM is responsible for this function. TRAM was demonstrated to directly interact with MyD88 in *in vitro* binding experiments in which a homotypic TIR-TIR interaction plays a vital role. The efforts to identify the interacting sites in the MyD88-TIR interaction revealed that two surface sites of MyD88-TIR are direct interfaces with TRAM-TIR. Interestingly, these interaction sites overlap with the sites for Mal binding [12]. Furthermore, cellular assays demonstrated the functional involvement of TRAM in the IL-18 signal transduction, and TRAM changed the localization of MyD88 from the cytosol to the membranous regions. These new findings strongly suggest that TRAM is the membrane-sorting adaptor for MyD88 in IL-18 signaling and plays a critical role in transmitting the signal. Thus, the mechanism of signal initiation is more conserved between the MyD88-dependent TLR4 pathway and IL-18 signaling than previously thought.

## Results

### Binding of TRAM to MyD88

**Figure 1:**
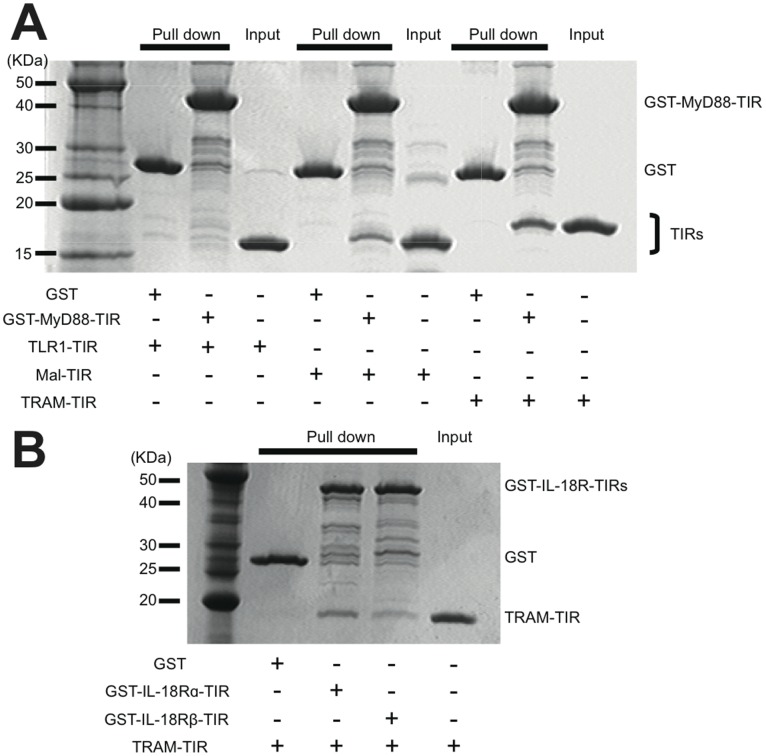
Protein interaction assays. (A) GST pull-down assay investigating the direct interactions between the GST-MyD88-TIR and the TRAM-TIR or TLR1-TIR. (B) GST pull-down assay between the GST-IL-18 receptor TIRs and TRAM-TIR.

**Figure 2:**
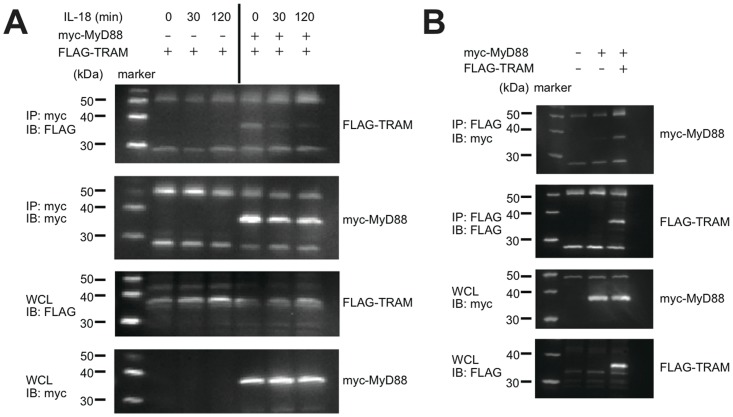
Assay of the interaction between MyD88 and TRAM in IL-18 signaling. (A) MyD88 and TRAM were co-expressed in HEK293T cells along with IL-18Rβ. IL-18 stimulation was carried out as indicated. MyD88 was immunoprecipitated using the Myc-tag antibody; co-immunoprecipitated TRAM was also detected. (B) The opposite direction co-immunoprecipitation assay between MyD88 and TRAM. Myc-MyD88 was detected with immunoprecipitated FLAG-TRAM.

Five TIR containing adaptor proteins have been previously identified: MyD88, Mal, TRIF, TRAM and SARM. Mal and TRAM were reported to be the sorting adaptors for MyD88 and TRIF, respectively [13]. Because a model structure of the TIR domain of TRAM represents a substantially large negatively charged surface area (Figure S1), while the TIR domain of MyD88 is covered by positive charge, we expected some interaction between these two adaptor molecules. We thus further hypothesized that TRAM also functions as the sorting adaptor for MyD88 in the IL-18 signaling pathway. To test these ideas, we first examined the direct interaction between MyD88 and TRAM. As both of the adaptors contain the TIR domain, which mediates the protein-protein interaction generally via homomeric or heteromeric TIR-TIR interactions, the direct interaction between the MyD88-TIR and the TRAM-TIR was examined with a GST-pull down assay. The results indicated that the wild-type MyD88-TIR directly bound the TRAM-TIR with a higher affinity than Mal, while the interaction between MyD88-TIR and TLR1-TIR, which had been shown not to bind the MyD88-TIR [14], was not detected in this method (Figure 1A). We then examined the interaction between MyD88 and TRAM in cells using a co-immunoprecipitation analysis. When Myc-MyD88 and FLAG-TRAM were co-expressed in HEK293 cells, the Myc-MyD88 constitutively associated with the FLAG-TRAM (Figure 2), which is consistent with our GST-pull down assay. Strikingly, upon stimulation of the cells with IL-18, the MyD88-TRAM complex gradually dissociated over a 30- to 120-minute time course. The HEK293 cells inherently express IL-18Rα (formerly called IL-1Rrp), which is a necessary component for IL-18 signaling, but lack IL-18Rβ (formerly called IL-1AcPL). Thus, we additionally co-expressed IL-18Rβ in the cells for this experiment.

### Binding of TRAM to IL-18 Receptors

For the activation of IL-18 signaling, the heterodimerization of two IL-18 receptors, IL-18Rα and IL-18Rβ, has been shown to be required [15]. These receptors belong to the IL-1 receptor superfamily and are thus structurally homologous to one another. The extracellular region contains immunoglobulin (Ig)-like chains, while the cytosolic region has a TIR domain that interacts with the TIR domain of MyD88. To test the interaction of TRAM with these IL-18 receptors, we performed GST pull-down assays using the TIR domains of the IL-18 receptors and TRAM. The results showed that the TIR domains from both IL-18Rα and IL-18Rβ directly bound the TRAM-TIR (Figure 1B).

### Involvement of TRAM in IL-18 Signaling

**Figure 3:**
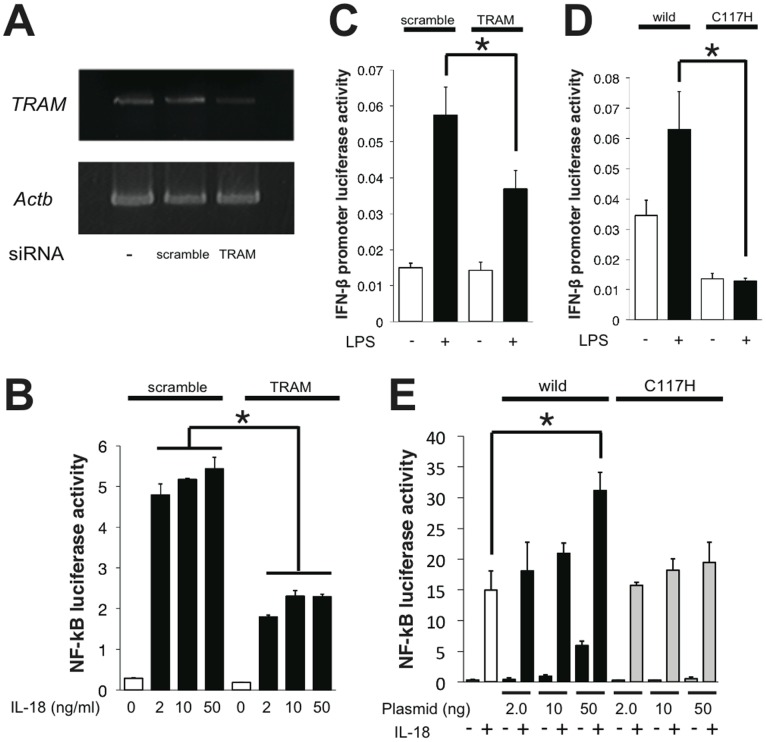
The knock-down effects of TRAM in IL-18 or LPS/TLR4 signaling. (A) RT-PCR of the *TRAM* mRNA knock-down in HEK293T cells by the psiRNA-TICAM-2 but not by the nonspecific scrambled sequence coded psiRNA vector. “-“ indicates cells not transfected with siRNA; “*Actb*” indicates the loading control (β-actin mRNA). (B–E) The effect of the knock-down of TRAM using shRNA or the dominant negative form of TRAM (C117H) for the IL-18 or LPS induced NF-κB or IFN-β-promoter luciferase activity assay. The NF-κB and IFN-β-promoter activities with IL-18 or LPS stimulation were significantly decreased in the IL-18Rβ co-transfected HEK293T cells or HEK293-hTLR4-MD2-CD14 cells.

**Figure 4:**
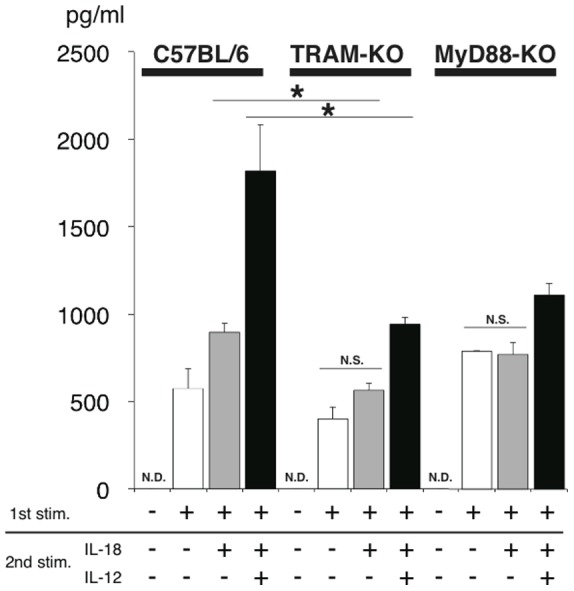
The IFN-γ production from IL-18 and/or IL-12 stimulated Th1 cells from TRAM-deficient mice and MyD88 deficient mice. The IFN-γ production levels were significantly reduced in TRAM deficient mice and MyD88 deficient mice. The black bars show the production levels from IL-18 and IL-12 co-stimulated Th1 cells, grey bars show those from IL-18 solely stimulated Th1 cells, and the white bars show those from no secondary stimulated Th1 cells.

After obtaining the evidence that TRAM interacts with MyD88 both *in vitro* and in cells, we then examined the possible involvement of TRAM in IL-18 signaling. We knocked-down the endogenous TRAM expression in HEK293 cells using siRNA techniques and then performed NF-κB reporter assays for the IL-18 signal transduction in the cells. The shRNA for the TRAM expressing vector was generated based on the previously reported target sequence [16]. When the expression of TRAM was knocked-down (Figure 3A), NF-κB activity after IL-18 stimulation was markedly decreased relative to the negative control experiments that used scrambled shRNA (Figure 3B). This result indicates that the knock-down of TRAM expression actually impaired the IL-18 signal transduction. We confirmed the knock-down effect of this shRNA sequence for TRAM via the LPS-stimulated activation of IFN-β promoter, which is presumably due to the suppression of the MyD88-independent TLR4 pathway mediated by TRAM and TRIF (Figure 3C). The effect was similar when compared to the results obtained from a dominant negative form of TRAM (C117H). TRAM (C117H) showed an almost complete shutdown of the LPS/TLR4/IFN-β signaling in the cells (Figure 3D) and the decrease of the enhancement of NF-κB activity induced by IL-18 (Figure 3E). To further confirm the involvement of TRAM in the IL-18 signaling pathway, we evaluated the cytokine production from helper type 1 differentiated T (Th1) cells isolated from TRAM-deficient mice and MyD88-deficient mice. IL-18 did not produce IFN-γ from not only MyD88-deficient Th1 cells but also TRAM-deficient Th1 cells, significantly. And, IL-18 alone or IL-18 and IL-12 co-stimulated TRAM-deficient Th1 cells produced significantly lower IFN-γ levels than those of wild-type Th1 cells (Figure 4).

### IL-18 Modulates MyD88-TRAM Subcomplex in Human Cells

**Figure 5:**
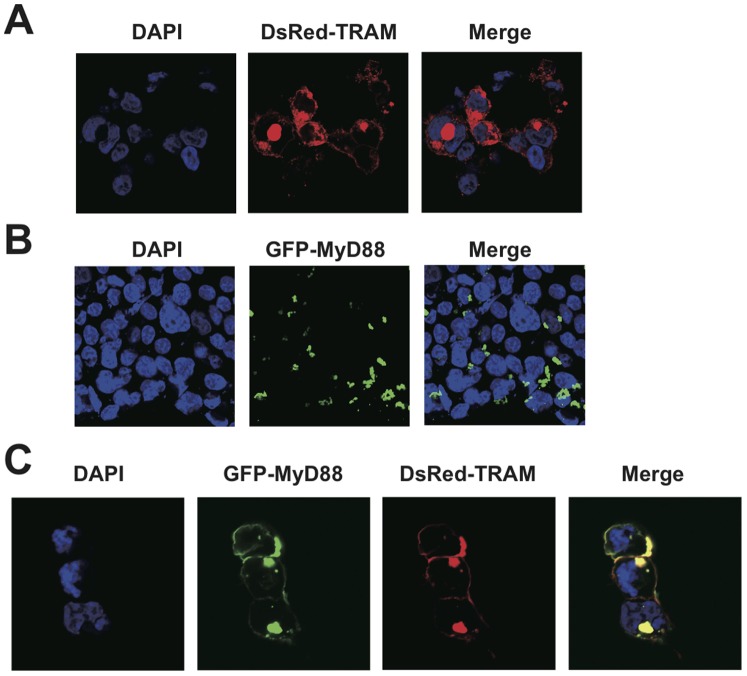
The localization of the MyD88 and TRAM complex in cells. (A–C) The localizations of the DsRed-TRAM (Red) and/or GFP-MyD88 (Green) in HEK293T cells. DAPI stained nuclei of HEK293T cells are shown in blue. Complexes of the DsRed fusion protein and GFP fusion protein are shown in yellow.

Next, we further investigated whether MyD88 actually interacted with TRAM in human HEK293T cells by live cell fluoroimaging. When we transiently transfected a DsRed-TRAM construct into HEK293T cells, the protein localized to the plasma membrane region, which was consistent with a previous report [10] (Figure 5A). In contrast, GFP-MyD88 was dominantly found as foci in the cytosol in the cells expressing the protein; this has also been shown by others [17] (Figure 5B). Strikingly, when HEK293T cells were co-transfected with expression plasmids for GFP-MyD88 and DsRed-TRAM, MyD88 proteins moderately co-localized with TRAM in the membrane regions (Figure 5C). These data strongly suggest that this transient interaction between the two proteins dramatically altered the localization of GFP-MyD88 from the cytosol to the membranes. Thus, TRAM both bound and endowed MyD88 with membrane targeting properties as has been previously demonstrated for Mal [5].

### Identification of Important Amino Acid Residues of the MyD88-TIR in IL-18 Signaling

**Figure 6:**
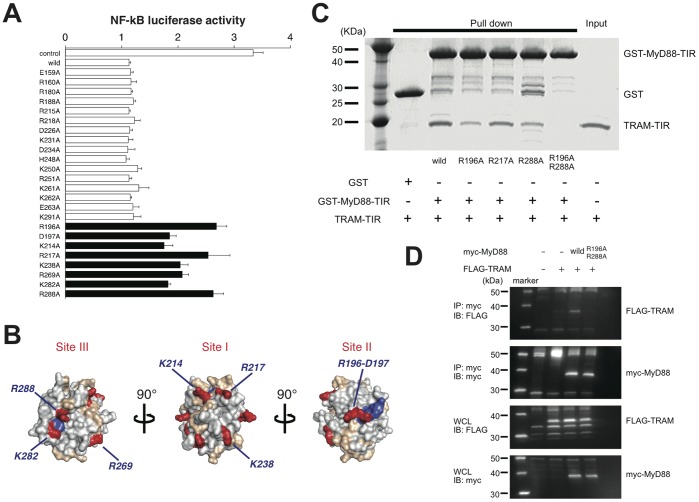
The interaction sites of MyD88 with TRAM. (A) Luciferase reporter gene activities with wild type and mutant types of the MyD88 TIR domain after IL-18 stimulation. The black bars indicate that the residues show significant difference with wild type. (B) The functional assays of IL-18 signaling presented on the 3D structure of the TIR domain of MyD88. Results of the functional assays are mapped onto the molecular surface of the MyD88 TIR domain. The amino acid residues judged to be significant by the luciferase assay are shown in red, while non-significant ones are shown in light brown. The conserved motifs of boxes 1–3 (FDA of box1, VLPG of box2, FW of box3) are shown in blue. (C) Assay to study the binding of the wild type or mutant TRAM TIR domain and MyD88 TIR domain. The representative alanine substitutions at Site II (R196A) or Site III (R288A) in MyD88 caused a reduced interaction with TRAM. The double alanine substituted mutant at Site II and Site III caused the complete abrogation of the interaction with TRAM. (D) Immunoprecipitation assay between MyD88 wild or R196A–R288A mutant, and TRAM.

Having established that MyD88 and TRAM directly interact and that the interaction is critical in IL-18 signaling, we next carried out experiments to identify the amino acid residues of MyD88 that are important in this interaction. A cell-based reporter assay system was utilized to examine various mutations in the MyD88-TIR as previously reported [12]. The results are shown in Figure 6A. An alanine substitution of any one of eight residues (Arg196, Asp197, Lys214, Arg217, Lys238, Arg269, Lys282 or Arg288) caused significantly reduced dominant negative inhibitory effects on IL-18 signaling indicating that these residues are involved in the signal transduction. These eight residues are mapped on the protein structure of the MyD88-TIR (PDB code: 2z5v) (Figure 6B). In our previous experiments for LPS/TLR4 signaling using the same luciferase reporter system, three discrete functional sites were found on the surface of the MyD88-TIR, which we designated Site I, Site II, and Site III (Figure 6B) [12]. Five out of the eight residues, Arg196 (Site II), Asp197 (Site II), Arg217 (Site I), Lys282 (Site III) or Arg288 (Site III) were previously found to be important in the LPS/TLR4 signaling. We then examined the direct binding of the representative mutants of each functional sites of MyD88-TIR to the TRAM-TIR using GST pull-down assays (Figure 6C). The results indicate that the binding between the MyD88-TIR and TRAM-TIR is dependent on Sites II and III because the alanine substitution of either Arg196 or Arg288 resulted in decreased binding. Furthermore, the interaction between the MyD88-TIR and TRAM-TIR was completely abrogated when both Arg196 and Arg288 were mutated. Additionally, co-immunoprecipitation assay also showed the reduction of interaction between MyD88 R196A-R288A mutant and TRAM (Figure 6D). In contrast, a Site I mutant, R217A, did not show a significant decrease in binding affinity. Overall, these interactions are very similar to those observed between the MyD88-TIR and Mal-TIR [12], indicating that TRAM and Mal share the same binding sites on MyD88-TIR.

## Discussion

### Involvement of TRAM in the IL-18 Pathway

Mal has been reported to function as the sorting adaptor in the TLR4 signaling that brings MyD88 to the plasma membrane to mediate the interactions between TLR4 and MyD88 [5]. Because the IL-1 and IL-18 receptors also form signal initiation complexes that contain MyD88, presumably at distinct membrane regions from TLR4, the existence of currently unidentified sorting adaptors that recruit MyD88 to specific membrane regions and mediate the interactions between MyD88 and the IL-1/IL-18 receptors has been hypothesized [5]. Although several reports have been published to date, the involvement of TRAM in IL-1 signaling remains controversial [16], [18], [19], [20]. Using TRAM-deficient mice, Yamamoto *et al.* showed that TRAM is not involved in IL-1 signaling. Despite the homology between IL-1 and IL-18, the relationship between TRAM and IL-18 had not yet been elucidated. In this study, we sought the sorting adaptor that acts in IL-18 signaling and found that TRAM fulfils this role. It was proposed that the electric potential is important for the specific interactions between the TIR domain proteins [21]. According to the electric surface potentials of the TIR domain structures, the TRAM-TIR and Mal-TIR both have a largely acidic surface patch, while the MyD88-TIR has a largely basic surface patch (Figure S1). Therefore we hypothesized that TRAM interacts with MyD88 and works as the sorting adaptor that recruits MyD88 in IL-18 signaling. In fact, TRAM has already been reported as the sorting adaptor in the MyD88-independent TLR4 pathway in which TRAM recruits TRIF to specific membrane regions [10], [22]. Nonetheless, in this work we obtained multiple results that indicate that TRAM functions as the sorting adaptor for IL-18 signals as we initially hypothesized. First, TRAM bound to MyD88 *in vitro* and in cells (Figure 1A, 2, and 5C). Second, the intracellular TIR domains of IL-18 receptors also bound to TRAM-TIR (Figure 1B). Finally, the shRNA knock-down of TRAM expression and knock-out of TRAM caused a significant decrease in the cellular response to IL-18 stimulation (Figure 3 and 4). These findings strongly suggest that TRAM functions as the sorting adaptor for MyD88 in IL-18 signaling. On the other hand, recent reports have shown that TRAM is associated not only with TLR4 but also with the TLR2 and TLR5 signaling pathways [23], [24]. These findings suggest that the conventionally accepted definition of the functions of the TIR domain-containing adaptor proteins in the TLR and IL-1/18 signaling pathways should be reconsidered.

### Time Dependent Change of the Interaction between MyD88 and TRAM

A particularly interesting feature we observed in this study was that the complex between Myc-MyD88 and FLAG-TRAM expressed in HEK293T cells decreased after IL-18 stimulation in a time-dependent manner (Figure 2). This observation suggests that either the rearrangement of the signal initiation complexes or the degradation of the components in the complexes is triggered by the activation of the IL-18 signaling. A similar rearrangement of the signal initiation complex has also been observed for the TLR4 pathway in a previous report; upon activation of TLR4 by LPS, TLR4 associated with MyD88 instantly, and the association was lost within 15 min [25]. Thus, such transient interactions between the receptors and adaptors and the subsequent loss of the interaction may be common to both the TLR and the IL-18 pathways especially because they utilize many of the same intracellular components. For the TLR4 complex, phosphorylation of TLR4 and Mal has been suggested to be involved in the rearrangements [25], [26]. TRAM has been shown to be phosphorylated by Protein kinase C-ε (PKC-ε) upon stimulation by LPS, and the phosphorylation has been implicated in regulating the myristoylation state and thus the membrane targeting [10], [27]. The membrane targeting of another myristoylated protein, MARCKS, has been shown to be regulated by phosphorylation; MARCKS is released from the plasma membrane upon the PKC mediated phosphorylation of a serine near its myristoylation site [28]. Similar to these examples, TRAM might be phosphorylated in the IL-18-induced dissociation of the MyD88-TRAM complex (Figure 2), although the mechanism underlying the dissociation and its relevance to signal regulation remain to be elucidated.

### The TRAM Interaction Sites of MyD88 are Similar to that for Mal

According to our previous study [12], Sites I, II, and III on the MyD88-TIR are functionally important in TLR4 signaling. These sites also were recognized to be important in IL-18 signaling (Figure 6A and 6B). We further demonstrated that Sites II and III act as the TRAM binding sites of the MyD88 TIR domain (Figure 6C), which overlap with the Mal binding sites [12]. The two sites are distantly located from each other on opposite molecular surfaces of the protein. Because of the molecular size of the TIR domain, it is unlikely that both sites present a simultaneous binding interface for a single TRAM-TIR to form a 1∶1 complex. It is more likely that each of these sites constitutes a distinct interface for a different TRAM molecule in different binding modes. Consistent with this hypothesis, a mutation of either Arg196 or Arg288 leads to only moderate losses in the binding to the TRAM-TIR in contrast to the fact that simultaneous mutations at both residues leads to a total loss of binding (Figure 6C and 6D). With this dual binding mode via Sites II and III, TRAM would be more efficient at recruiting MyD88 to membrane regions. It should be noted that this dual binding mode was also found in the binding between the MyD88-TIR and Mal-TIR [12], which implies that this sort of multiple binding mode is common to the TIR-containing adaptor proteins.

Additionally, in human, the deficiencies of TIR domain containing adaptors, MyD88 and TRIF, have been recently reported [29], [30]. These deficiencies were categorized into the innate immune defects. One of the mutations of MyD88, R196C, is known to cause the severe pyogenic bacteria infection due to the loss of interaction between TLR2, Mal and MyD88 [12]. According to the above-mentioned results, Arg196 is one of the binding sites of MyD88 to TRAM. Therefore, the substitution of Arg196 may abrogate not only an initial signaling of TLR mediated by the interaction between Mal and MyD88 but also a secondary enhancement of immune responses mediated by IL-18 induced interaction between TRAM and MyD88 in T cells or NK cells for etiology of human MyD88 deficiency syndrome.

In summary, we have established an unexpected connection between TRAM and IL-18 signaling, which is mediated by a direct TIR-TIR interaction between MyD88 and TRAM, and we proposed that TRAM is the sorting adaptor for IL-18 signaling. Based on the results obtained in this study, we present a schematic model for signal initiation from activated IL-18 (Figure S2) that is similar to the model for the LPS/TLR4 system.

## Materials and Methods

### Vector Preparations

The following recombinant protein expression cassettes were subcloned into pGEX4T-1, pGEX5X-1 or pGEX5X-3 (GE Healthcare, Buckinghamshire, England): IL-18, IL-1β, MyD88-TIR (amino acid residues 148–296), TRAM-TIR (66–235), TLR1-TIR (625–786), IL-18Rα-TIR (374–541), and IL-18Rβ-TIR (407–599). A cDNA encoding the MyD88 TIR domain tagged at the N-terminus with a Myc-epitope was cloned into the plasmid vector pcDNA3.1+ (Invitrogen, California, USA). IL-18Rβ and TRAM constructs tagged at the C-terminus with an AU1- or FLAG-epitope, respectively, were also cloned into pcDNA3.1+. Mutants of TRAM and the TIR domain of MyD88 were generated using the GeneEditor *in vitro* Site-Directed Mutagenesis System (Promega, Wisconsin, USA). A pGL3-Basic Vector (Promega) containing four κB binding sites, which was used in the NF-κB luciferase reporter assay, and a Renilla luciferase reporter vector used as an internal control in the assay were gifts from Dr. Sewon Ki and Dr. Tetsuro Kokubo (Yokohama City University). An IFN-β promoter region sequence containing pGL4-Luc (Promega) was generated. A pAcGFP-C1-MyD88 (GFP-MyD88) and a pDsRed-Monomer-N1-TRAM (DsRed-TRAM) were also generated (Takara Bio, Shiga, Japan).

### Protein Expression and GST Pull Down Assay

The TIR domain of the MyD88 wild type and mutants (R196A, R217A, R288A, and R196A–R288A) and the IL-18 receptors (Rα and Rβ) were purified as GST (glutathione S-transferase) fusion proteins according to methods previously described (1). The TIR domain of human TRAM and TLR1 was also obtained by a similar procedure as previously described for the MyD88-TIR. These purified proteins were incubated with Glutathione Sepharose 4B (GE Healthcare) for three hours at 4°C, and then these resins were washed four times with wash buffer (20 mM potassium phosphate buffer (pH 6.0), 100 mM KCl, 0.1 mM EDTA, 10 mM DTT, and 0.5% Triton X100), and then analyzed by SDS polyacrylamide gel electrophoresis with Coomassie Brilliant Blue staining. Experiments were performed in triplicate. The mature form of human IL-18 and IL-1β were prepared using E.Coli expression system according to previously reported methods [15].

### Cell Culture

HEK293-hTLR4-MD2-CD14 cells were purchased from Invivogen (California, USA), respectively. HEK293 cells were cultured in Dulbecco’s Modified Eagle Medium (high glucose-containing D-MEM, Invitrogen) supplemented with 10% heat-inactivated fetal bovine serum (SIGMA-ALDRICH, Missouri, USA), penicillin (100 U/mL) and streptomycin (100 µg/mL). All cells were incubated at 37°C in a humidified atmosphere of 5% CO2. The splenic pan T cells were isolated using Pan T Cell Isolation Kit II (Miltenyi Biotec, Bergisch Gladbach, Germany) from the spleen of TRAM-deficient mice, MyD88-deficient mice and background mice (C57BL/6) supplied by Oriental Bio Service (Kyoto, Japan). The purified splenic T cells were incubated with or without 2 ng/ml recombinant murine IL-12 (p70) (PEPROTECH, New Jersey, USA) on BIOCOAT anti-mouse CD3 T-cell activation plates (BD Biosciences, Massachusetts, USA) in order to be differentiated into Th1 cells. After 4 days of culture, T cells were washed and restimulated with 20 ng/ml recombinant murine IL-18 (MBL, Nagoya, Japan) and/or 2 ng/ml recombinant murine IL-12 (p70) on anti-mouse CD3 T-cell activation plates for 24 hours. All animal experiments were carried out in accordance with the NIH Guide for Care and Use of Laboratory Animals. These cells were cultured in RPMI1640 media (Invitrogen) supplemented with 10% heat-inactivated fetal bovine serum, penicillin (100 U/mL) and streptomycin (100 µg/mL).

### Co-immunoprecipitation Analysis

HEK293T cells in 100 mm plates were transfected with 5.0 µg of pcDNA3.1+ IL-18Rβ, 5.0 µg of pcDNA3.1+ Myc-tagged MyD88 (full-length) wild or R196A–R288A mutant and/or 5.0 µg of pcDNA3.1+ FLAG-tagged TRAM (full-length) using Lipofectamine 2000 (Invitrogen). After 18 hours, the culture media were replaced. After 24 more hours, the cells were incubated with or without IL-18 (10 ng/mL). These cells were washed with cold PBS and harvested with cell lysis buffer (Tris-HCl buffer (pH 7.5) with 10 mM NaCl, 10 mM EDTA, 0.5% Triton-X100, a protease inhibitor cocktail (Roche Diagnostics, Mannheim, Germany), and a phosphatase inhibitor cocktail (PIERCE, Illinois, USA). The soluble cell lysates including 1000 µg protein were incubated with 5 µg of anti-Myc antibody (Invitrogen) or anti-FLAG M2 monoclonal antibody (SIGMA-ALDRICH) for 60 minutes; 50 µl of MultiMACS Protein G MicroBeads (Miltenyi Biotec) that had been equilibrated with cell lysis buffer for 30 minutes at 4°C was then added to the lysates. After incubation, the immune complexes were applied to the magnetic columns. The protein complex samples were then solubilized with 1× Laemmli sample buffer after four washes with wash buffer. The samples were analyzed by western blots using an anti-Myc antibody and an anti-FLAG M2 monoclonal antibody.

### Knock-down with shRNA or Dominant Negative Mutant of TRAM

The shRNA expression vector psiRNA-h7SKgz-Scr (used as a negative control because it contained a scrambled sequence) and psiRNA-TICAM-2 were purchased from Invivogen. For the reporter gene assays, HEK293T or HEK293-hTLR4-MD2-CD14 cells were seeded at a density of 2.0×10^5^ cells/mL per well in a 96-well plate. These cells were transfected with or without pcDNA3.1+ IL-18Rβ-AU1, NF-κB luciferase reporter vector, and Renilla luciferase reporter vector with either the psiRNA-h7SKgz-Scr or psiRNA-TICAM-2, pcDNA3.1+ TRAM-FLAG wild or C117H mutant vector using Lipofectamine 2000. After 18 hours, the culture media were replaced with fresh medium, and after an additional 24-hour incubation, the culture media were replaced with fresh medium containing recombinant human IL-18 (2.0, 5.0, 50.0 or 10.0 ng/mL) or LPSO127: B8, which is derived from *E. Coli* strain (100 ng/mL) (SIGMA-ALDRICH), incubated for 6 hours. The luciferase reporter gene activities were analyzed using a Dual-Luciferase Reporter Assay System (Promega). The statistical significance of the differences in the luciferase activities was determined using Dunnett’s multiple comparison test. The statistical significance was assigned to be *P<*0.05.

### RT-PCR

Total RNA from cells seeded in six-well plates was isolated with ISOGEN (Nippon Gene, Toyama, Japan) according to the manufacturer’s instructions. Reverse transcription was performed with a 1st Strand cDNA Synthesis Kit (Roche Diagnostics) according to the manufacturer’s instructions. The cDNA obtained was used in PCR with Taq DNA polymerase (Toyobo, Osaka, Japan) to determine the relative amount of TRAM mRNA.

### ELISA

Culture supernatants in test tubes were centrifuged to remove the cells and then stored at –80°C until analysis. The IFN-γ concentrations were measured using a Mouse IFN-γ Quantikine ELISA Kit (R&D Systems, Minnesota, USA). The statistical significance of the differences in the cytokine productions between the wild type cells and the TRAM or MyD88 deficient cells was determined using two-way ANOVA with Bonferroni’s multiple comparison test. The statistical significance was assigned to be *P<*0.05.

### Confocal Microscopy

For direct immunofluorescence, HEK293T cells co-transfected GFP-MyD88 and DsRed-TRAM were washed in phosphate-buffered saline and fixed for 10 min in 4% paraformaldehyde in phosphate-buffered saline. Cells were then permeabilized with 0.2% Triton X-100 in phosphate buffered saline for 10 min at room temperature. Samples were mounted onto coverslips with Pro-Long Gold Antifade reagent (Invitrogen) and were examined on a Zeiss LSM5 EXCITER confocal microscope. All images were acquired using an aplan-Apochrom at 63X with a 1.4-N.A. objective or at 100X with a 1.4-N.A. objective.

### The Screening Method of Functional Residues of MyD88-TIR in IL-18 Signaling

HEK293T cells were transfected with pcDNA3.1+ control vector or pcDNA3.1+ myc-MyD88 TIR domain (wild-type or mutants), pcDNA3.1+ IL-18Rβ-AU1, NF-κB luciferase reporter vector, and Renilla luciferase reporter vector using Lipofectamine 2000 according to the manufacturer’s instructions. These transfectants were stimulated with recombinant human IL-18 (100 ng/mL) for 6 hours. The luciferase reporter gene activities were also analyzed using a Dual-Luciferase Reporter Assay System (Promega). The statistical significance of the differences was determined using Dunnett’s multiple comparison test. The statistical significance was assigned to be *P<*0.05.

## Supporting Information

Figure S1 **The surface electrostatic potential of the TIR domain structure models from the TIR domain containing adaptor proteins.** These structure models were predicted from the template structure of the MyD88-TIR (PDB code: 2z5v) using Discovery Studio 2.6 software (Accelrys). TRAM and MAL have a largely acidic surface patch, while MyD88 has a largely basic surface patch.
(TIFF)

Figure S2 **A schematic model of the two distinct regulation patterns in LPS induced TLR4 signaling and IL-18 signaling.** MyD88 is efficiently delivered to receptor specific membrane regions by the membrane binding activities of the two associated molecules of Mal or TRAM so that it can form signal initiation complexes with activated TLR4 or IL-18 receptors. Upon stimulation, MyD88 starts to transmit signals through interactions with activated TLR4 or IL-18R, which triggers the phosphorylation cascade mediated by IRAKs and TRAF6.
(TIFF)
