# Supplementary figures and images for: TRAM Is Involved in IL-18 Signaling and Functions as a Sorting Adaptor for MyD88
Source: PLoS One. 2012 Jun 7;7(6):e38423. doi: 10.1371/journal.pone.0038423 (PMC3369926; doi:10.1371/journal.pone.0038423)

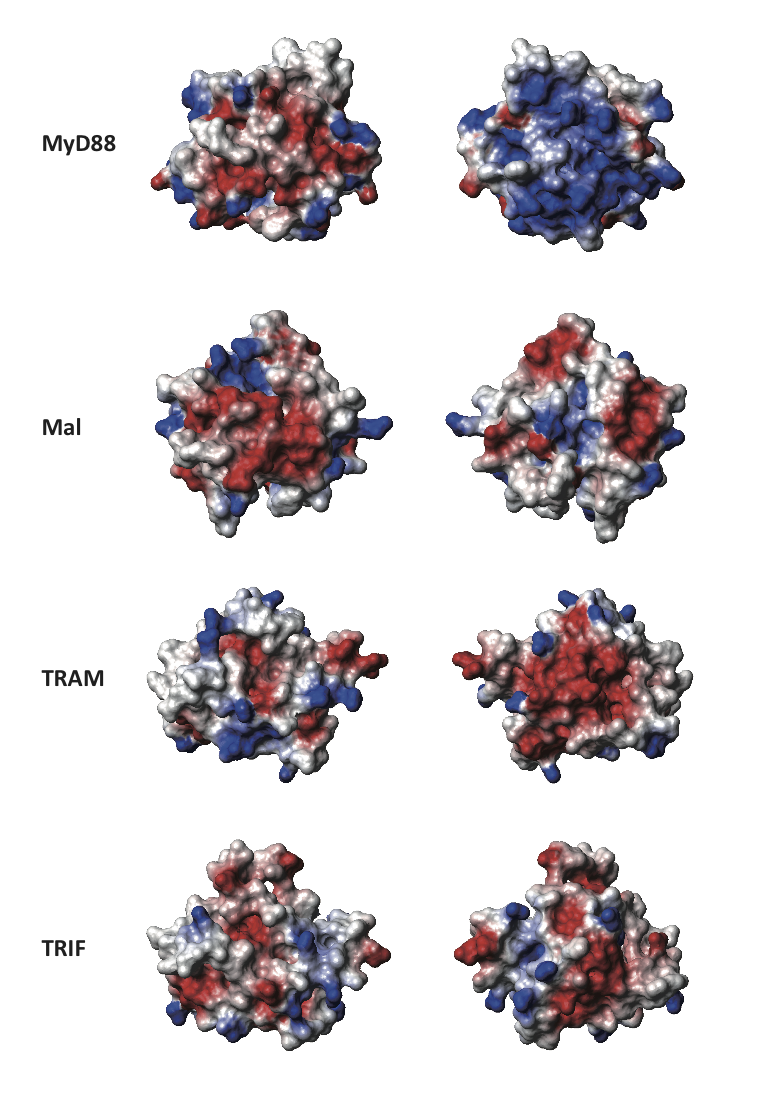

Supplement: Figure S1 — The surface electrostatic potential of the TIR domain structure models from the TIR domain containing adaptor proteins. These structure models were predicted from the template structure of the MyD88-TIR (PDB code: 2z5v) using Discovery Studio 2.6 software (Accelrys). TRAM and MAL have a largely acidic surface patch, while MyD88 has a largely basic surface patch. (TIFF) [file pone.0038423.s001.tiff]

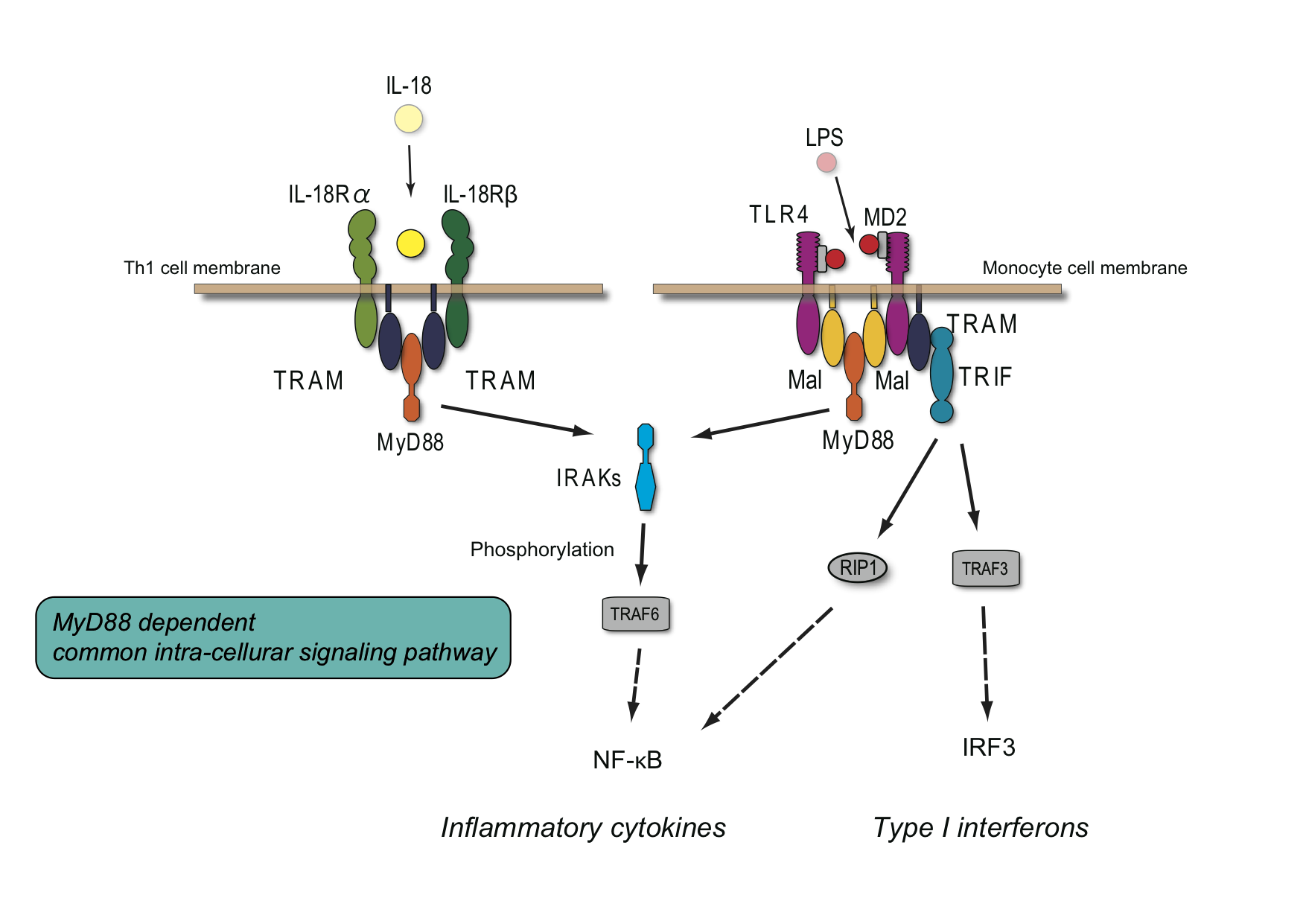

Supplement: Figure S2 — A schematic model of the two distinct regulation patterns in LPS induced TLR4 signaling and IL-18 signaling. MyD88 is efficiently delivered to receptor specific membrane regions by the membrane binding activities of the two associated molecules of Mal or TRAM so that it can form signal initiation complexes with activated TLR4 or IL-18 receptors. Upon stimulation, MyD88 starts to transmit signals through interactions with activated TLR4 or IL-18R, which triggers the phosphorylation cascade mediated by IRAKs and TRAF6. (TIFF) [file pone.0038423.s002.tiff]
